# Role Models and Teachers: medical students perception of teaching-learning methods in clinical settings, a qualitative study from Sri Lanka

**DOI:** 10.1186/s12909-016-0576-6

**Published:** 2016-02-09

**Authors:** Vathsala Jayasuriya-Illesinghe, Ishra Nazeer, Lathika Athauda, Jennifer Perera

**Affiliations:** Daphne Cockwell School of Nursing, Ryerson University, 350 Victoria St, Toronto, ON Canada M5B 2 K3; Teaching Hospital, Karapitiya, Sri Lanka; Faculty of Medicine, University of Kelaniya, Kelaniya, Sri Lanka; Faculty of Medicine, University of Colombo, Colombo, Sri Lanka

**Keywords:** Clinical teaching, Role modeling, Medical education, Asia, Sri Lanka

## Abstract

**Background:**

Medical education research in general, and those focusing on clinical settings in particular, have been a low priority in South Asia. This explorative study from 3 medical schools in Sri Lanka, a South Asian country, describes undergraduate medical students’ experiences during their final year clinical training with the aim of understanding the teaching-learning experiences.

**Methods:**

Using qualitative methods we conducted an exploratory study. Twenty eight graduates from 3 medical schools participated in individual interviews. Interview recordings were transcribed verbatim and analyzed using qualitative content analysis method.

**Results:**

Emergent themes reveled 2 types of teaching-learning experiences, role modeling, and purposive teaching. In role modelling, students were expected to observe teachers while they conduct their clinical work, however, this method failed to create positive learning experiences. The clinical teachers who predominantly used this method appeared to be ‘figurative’ role models and were not perceived as modelling professional behaviors. In contrast, purposeful teaching allowed dedicated time for teacher-student interactions and teachers who created these learning experiences were more likely to be seen as ‘true’ role models. Students’ responses and reciprocations to these interactions were influenced by their perception of teachers’ behaviors, attitudes, and the type of teaching-learning situations created for them.

**Conclusions:**

Making a distinction between role modeling and purposeful teaching is important for students in clinical training settings. Clinical teachers’ awareness of their own manifest professional characterizes, attitudes, and behaviors, could help create better teaching-learning experiences. Moreover, broader systemic reforms are needed to address the prevailing culture of teaching by humiliation and subordination.

## Background

Contemporary discourses around medical graduates’ lack of communication skills, ethics, and professionalism takes a critical view of the teaching-learning strategies, methods, and techniques used and the role of the clinical teachers in developing these skills. While a growing body of work originating in North American, European, and British Medical Schools contribute to this discussion [1–5], there are knowledge gaps about clinical teaching-learning methods used in other parts of the world, especially the global south.

Asia is home to a large number of medical schools producing doctors and nurses to meet the demands of an ever-increasing health workforce [6, 7]. A global call for uniform standards in medical education has encouraged many medical schools in Asia to review their study programs and to keep abreast of changes in other parts of the world. The introduction of medical education units, curricular reviews, and an increased interest in adopting evidence-informed teaching-learning methods are some of the resultant positive changes in this region [7–9]. However, lack of locally-relevant, empirically-sound, medical education research that can guide these reforms is of concern [10]. For example, only 1 % of articles published in *Academic Medicine* and 8 % of articles published in *Medical Education*, between 1995 and 2000, were based on settings in Asia [11]. Promoting medical education research and bridging the gap between research and practice are crucial areas of concern for medical schools in Asia.

### Medical education in the local context

Medical schools in Asia have colonial roots and a long history of education shaped by British, French or North American systems [7, 12]. The extant medical education systems have been criticized as traditional, didactic, and ‘old fashioned’ [7]. Sri Lanka, the setting for this study, is a south Asian island nation with a similar long history of Medical education; the Colombo medical school was founded in 1870 [13]. Unlike the extensive private medical school systems in India, Malaysia, and Singapore [8], medical schools in Sri Lanka are predominantly public Universities, with the exception of one recently-established private school. Eight state-managed Universities offer undergraduate and postgraduate courses in Medicine. Almost all of these medical schools have undergone curricula review and revisions aiming to shift away from traditional didactic methods, advocating for student-centered, integrated curricular and teaching-learning approaches [13, 14]. There are also attempts to include community-based modules and behavioral streams aimed at developing communication skills, ethics, and professionalism. However, majority of these changes have less of a focus on teaching-learning methods used in clinical settings, particularly lacking attention to methods used during the final year which predominantly consists of clinical training. Moreover, the existing small body of medical education research in Sri Lanka also focusses on non-clinical aspects of training. An article written by a medical student from United Kingdom about his observations during an elective placement at a teaching hospital in Sri Lanka provides some insights into a hierarchical, teacher-centered system of education [15]. He also noted fear, apprehension, and lack of confidence among the students because of strict and authoritarian teaching methods used by the clinical teachers.

This explorative study aims to describe medical students experiences in 3 medical schools in Sri Lanka to provide a nuanced, in-depth understanding about the teaching-learning methods used in clinical training settings.

## Methods

After obtaining research ethics board approval from the ethics review committee of the University of Sri Jayewardenepura, we conducted informal interviews with medical graduates and clinical teachers. These discussions helped to identify the most appropriate data collection method (interviews), to develop data collection instruments (interview guides), and to address some of the challenges to participant recruitment. For example, individual interviews were chosen as the preferred data collection method over focus groups because students indicated that their peers may not volunteer for group discussions and may not be comfortable to share personal stories about learning experiences in a group setting. We invited recent graduates from 3 medical schools for individual interviews. General information about the study was shared with potential participants through peers. Those who contacted a research assistant and volunteered to join the study were informed verbally and via the consent form of the voluntary nature of participation, their right to refuse to participate or answer any specific questions, and/or to end the interview at any time. Participants met with a trained female research assistant to sign consent and arrange an interview. The interviews were conducted in English using a semi-structured interview guide, at a private location chosen by the participant. Interviews were audio-recorded with consent of the participants and each interview lasted about 1–2 h.

Literature reviews and information gathered through informal interviews helped to develop the initial interview guide, which was kept open to allow emerging findings to be clarified in later interviews.

The authors’ institutional affiliations at the time of data collection as academic faculty members in 2 medical schools in Sri Lanka, was relevant for data collection and analysis in this study. To avoid this being perceived as coercive, these authors avoided contact with study participants, and their names were not included in information sheets and consent forms (with ethics review committee approval) to avoid their names being published and/or associated with this study,. However, to allow participants to make an informed decision, those who were ready to sign consent were verbally informed about the researchers conducting the study. They were allowed to take as much time as necessary before deciding whether they want to join the study, and their decision to participate or not was only known to the research assistant. None of the participants withdrew, postponed, or ended an interview prematurely.

Data analysis coincided with the interviews allowing the researchers to gather interviews until data saturation occurred. After each interview, research assistants listened to the audio-recordings and removed any names of persons or places mentioned by recording an empty segment over these sections. This ensured confidently and anonymity for the participants and also removed the possibility of the authors identifying any of the individuals referred to in the interviews. After removing identifying information, the research assistants transcribed the interviews verbatim, and provided text and the audio files to the authors to do random checks for accuracy. Using a sentence-by-sentence process, each interview was read/re-read, meaning units identified, coded and sorted into categories using inductive qualitative content analysis method. An open coding scheme was used for the initial interviews, and after achieving consensus among the authors, this coding frame was used to code the remaining transcripts. Two authors independently applied this coding to the transcripts and compared for consistency, any disagreements were resolved by discussion. Commonalities and variations were identified across all interviews before re-grouping the codes into three broad categories. An example of one such category is shown in Table 1 below. This process of categorization involved several rounds of discussion between the two authors.

**Table 1:** Example of code grouping into a category

Category	Characteristics of teaching-learning experiences	
Sub category	Role modelling	Purposive teaching
Codes	Following them around No ‘teaching’ Haphazard teaching Waste of time Absence of teachers No mutual respect Confusion Avoidance Discordance with expectations	Pre-planned Prepared Well Organized Dedicated time Frequent contact Participatory Mutual respect Clarity

### Reflection on the methodology and trustworthiness of the study

The first and fourth authors’ insider status was relevant for this study, because the research focused on their own social group [16]. However, as members of the para-clinical faculty who did not directly work with final year students, they were somewhat detached from the clinical teaching-learning environments within hospitals. This ‘limited’ insider status, none the less, enabled the authors to situate the data within the broader academic milieu. This was useful during data analysis because contextual information about the places and persons referred to during interviews was removed from the recordings and not available to the authors.

## Results

Twenty eight recent medical graduates were interviewed 3–6 months after graduation from their respective medial schools, 18 of them were females. The number of students from each university were, more or less, equally divided (10 each from 2 universities, and 8 from the third). The 3 medical schools were affiliated with 3 universities (which will not be named here) and are governed by the University Grants Commission of Sri Lanka. The commission establishes criteria for admissions, the length and overall structure of the study program, as well as the timing and nature of assessments. All of the participants in our study belonged to one cohort of graduates who had been admitted into Universities under these uniform entry criteria.

According to the participants their final year of education consisted mostly of ward-based clinical training at various specialties and sub-specialties. Each appointment was 4–8 weeks long, and students were ‘attached’ to one or more consultants in the unit during this time. Students had access to learning objectives and log books in some of the attachments.

The 3 emergent categories from the data: characteristics of teaching-learning experiences (role modelling and purposeful teaching), characteristics of the teacher (‘figurative’ role models and ‘true’ role models), and the reciprocal interactions (‘avoid getting caught’ and ‘something to take away’) are described below.

### The teaching-learning experience

#### Role modeling

Role modeling has been described as ‘the process by which faculty members demonstrate clinical skills, model and articulate expert thought processes, and manifest positive professional characteristics’ [Irby cited in 3]. The teaching-learning experiences described by participants showed characteristics of role modelling; students were expected to be present while teachers conducted ward rounds, theatre work, or clinic sessions. Students described spending most of the time observing teachers while they carry out their usual clinical work, waiting for ‘teachable’ moments to interact directly with them. This is characteristic of role modelling where students spend most of the time observing teachers, however, because the periods of direct interactions were few and/or brief, students felt they were not ‘learning anything.’ Most of the time spent in observing teachers was perceived as a ‘waste of time’ because teachers did not dedicate time towards what students considered to be ‘teaching’ activities.

*“Sometimes we walk with the consultant during ward rounds for 3–4 hours and they say about 4 words to us, they could tell us to vanish off, they don’t, they just let us stay and don’t teach a word”(respondent 2).*

Lack of dedicated time towards teaching meant lack of opportunities for students to seek clarifications and/or discuss concerns.

*“[A] comes once-in-a-while, visited 5 times in those 8 weeks. She was teaching us how to take a history and examination, I didn’t understand it, if she really wants to teach she should come more often” (respondent 21).*

The most common strategies used for instruction and to provide feedback were ‘questioning’ and ‘commenting’ aimed at assessing completeness of a clinical history, ability to recall factual information, or skills in eliciting clinical signs. Teachers used comments or statements to provide feedback about the students’ performance, but this was not seen as facilitating learning. The feedback often created confusion for the students during what they perceived to be haphazard teaching-learning sessions.

*“[B]’s teaching is great ! (sarcasm), she taught us the management of Sepsis in 30 seconds, made us wait till 12 midnight, and taught us in 30 seconds”(respondent 16).*

*“She tells us ‘show me how you do a XXXX examination, and afterwards tell us ‘you don’t know anything, that is not how you do it’, but she never tells us the correct way to do it.”(respondent 11)*

### Purposeful teaching

Teaching or mentoring is considered a more formal interaction with a teacher purposefully facilitating student learning through specific methods of instruction [17]. Participants described pre-planned organized teaching sessions referred to as ‘ward classes’, which could be categorized as purposeful teaching. The ‘ward classes’ were perceived as well-organized and useful, because dedicated time was available for students to engage in discussions and/or clarify issues. As one student explains below, being able to sit down during a ‘class’, helped to focus on the lesson; these sessions was perceived as being more effective than haphazard ‘ward round’ teachings.

*“[C] had classes with us sitting down and would discuss in detail. It was not a class where you had to stand for 2 hours in the ward and the consultant is sitting with his legs up on the table not caring that you are about to faint” (respondent 18).*

Because they were scheduled sessions with prior identification of topics for discussion, students were able to prepare in advance for the teaching-learning activity and this helped create a positive experience.

*“By the end of the ward class we were happy about what we have leant, not just relieved that it [teaching session] was over, there was something left after the class” (respondent 12).*

### Characteristics of the teachers

The teachers’ characteristics could be described as ‘figurative’ role models and ‘true’ role models.

#### The ‘figurative’ role models

Participants described majority of their teachers as rude, arrogant, and condescending. They appeared to be ‘figurative’ role models, because even though they were aiming to ‘role model’ they did not manifest positive professional characteristics [3]. They were not present in the teaching-learning environment frequently or long enough for the students to be able to observe modeled behavior.

*“There was no one in ward [X] especially consultants, they just come and to go, there is no one to teach, no time to interact with them. They should be more approachable” (respondent 7).*

Even when they were interacting with students, ‘figurative’ role models often failed to model professional characteristics, and as a result they were seen as hostile, intimidating, sexist, and discriminatory towards students.

*“Some of the consultants are very hostile, it’s take me a while to think of someone who didn’t make me feel that way. Some people used ‘miss’ [to address students] in a very condescending way, some were condescending anyway, you are condescended upon, you are made to feel like a really tiny person, a useless person” (respondent 11).*

*“Then there are these consultants who always say sexually offensive jokes, I think it is supposed to be funny, but it was very embarrassing and I was wishing I’d never have a class with him again” (respondent 3).*

*“You are the ones that provide them with entertainment. Maybe in this country ridiculing is one of the wonderful [sarcasm] ways of getting people to learn, the worst was getting humiliated in the ward round”(respondent 18).*

### The ‘true’ role models

‘True’ role models, in contrast, showcases positive professional characteristics. They were described as excellent teachers, astute leaders, and caring clinicians who were regularly present and dedicated time for purposeful teaching-learning activities. Students described opportunities to observe good bed-side manner, excellent communication skills, and an empathic attitude towards patients in the presence of these teachers.

*“[D] was a very good clinician and his teaching was excellent, maintained the ward in aproper manner” (respondent 8).*

‘True’ role models encouraged students with responsive feedback, and treated students as professionals.

*“[E] is competent, compassionate and caring, I see all 3 qualities in him, he comes at 7.00 am, we present the histories, examination and investigation findings, not the house officer, so he gave us responsibility, even during holidays he comes and teaches till noon, even during surgery he teaches us, treats us like a colleague (respondent 7)*

*“Very kind hearted, won’t scold us for any answer that we give, even when we say something foolish he’ll say ‘I know you know the answer, you just have to think and put it into words’. His personality, approach, teaching method, patient approach, way of analyzing symptoms, are all done well, I like him very much”(respondent 12).*

### The reciprocal interactions

Third emergent category points to the students’ reciprocal interactions in the teaching-learning environment, revealing 2 reactions, ‘avoid getting caught’ and ‘something to take away’.

#### ‘Avoid getting caught’

‘Avoid getting caught’ characterizes a common recurring theme in students’ responses to role modelling and ‘figurative’ role models. Majority of students avoided interactions with ‘figurative’ role models. For example, they tried to stand at the back of the group and avoided speaking-up. Their main focus was on acting subservient, because they perceived this was expected of them, hoping that they will not draw attention that could result in retribution, punishments or humiliation; in their own words ‘*trying to avoid getting caught’*.

*“Everyone [dragged out] starts standing at the back, and I would stop listening to what is going on. [G] even wants values of reports, so the whole time I am memorizing, hiding behind others. So that was pretty negative. You don’t learn anything” (respondent 12).*

*“We take histories to get away from the person doing the ward round. From 8 am to noon, the focus is on trying to get away with your history during the ward rounds. I try to be invisible, not draw attention, I am scared of those who scold, actually I cut [avoid] classes. I try to survive for the time, to make sure they won’t catch me” (respondent 3).*

The avoidance methods were often reactions to previous negative interactions with these teachers, because some were known to verbally and/or physically harass students or to punish them in unfair ways, for example, they could be made to gather large numbers of patient histories as described below.

*“Some [teachers] were really traumatizing the students, there were some who would physically harass almost every student in the group, whether you gave a correct or wrong answer, you get hit on the head, he does it to girls, it was the worst period of my clinical life” (respondent 4).*

*“We have to be prepared with the history, sometimes we have to take histories of all the patients in the wards, sometimes we take 40 histories as a punishment” (respondent 5).*

### ‘Something to take away’

In contrast ‘something to take away’ describes students’ positive interactions with ‘true’ role models. These interactions happened during both types of interactions, role modeling and purposeful teaching. Students recognized being given responsibility and felt respected by the teachers. This was reciprocated with respect and an enthusiasm to participate in the teaching-learning activities resulting in positive learning experiences. One student describes this as being able to ‘*remember every single word taught by [H]’*. During these interactions students describe a sense of self-efficacy and self-esteem because they were not made to feel incompetent or subordinate.

*“[H] treated us like fellow junior doctors, didn’t treat us like ‘oh my god you are all stupid medical students’. He talked to us like professionals and addressed us by our name. ‘Tell me what would you do if this was your patient, OK, I agree what you say makes sense, but how would you do it in the ward ?’ With him it would be a professional discussion, not something where I’d say something and they’d go ‘Whaat ??? are you crazy ? Oh my god what have you been doing all this time’ that would make me feel really stupid and I would NEVER answer again. It wasn’t like that with [H], it was all very professional. Made me feel like a professional, not like a silly girl, which is how I felt most of the other times”(respondent 3).*

## Discussion

There is a lack of clarity and consensus about the clinical teacher’s role in creating effective teaching-leaning experiences that can contribute towards clinical and professional skill development in medical students [18]. Confusion about these different roles has some researchers calling for a distinction between role modeling and teaching [17]. They consider role modeling to be separate from mentoring or teaching, because teachers are not seen as purposefully facilitating student learning when they are role modeling [3, 17].

Among the 28 participants of our study we observe a similar confusion around role modeling; when academic members were ‘role modeling’ this was discordant with what students expected to constitute clinical ‘teaching’. Most of the clinical teaching-learning experiences described across the 3 medical schools were similar and constitute mostly of role modeling. Clinical teachers expect students to observe their behavior while professionally conducting themselves. Students were expected to be present for ward rounds, clinics, and theater sessions to observe clinical skills, bed side manner, and other professionals skills, with an assumption that the clinician is modeling ideal behavior that is to be ‘learned’ by the students through observation. As Sandhu and others [17] describe, clinical teachers tend to consider role modeling to be synonymous with teaching. However, students perceived this as haphazard unstructured teaching where they spend large amounts of time ‘following them around’. There appears to be emphasis on rote-learning and factual recall with little room for application, analysis, and evaluation of knowledge.

Furthermore, teachers who mostly ‘modeled’ behavior without creating dedicated space and time for students were not perceived as ‘true’ role models; they were seen as manifesting unprofessional characteristics during their brief periods of interactions with students. As described elsewhere [17, 18], role modeling or behaving ‘professionally’ did not directly transform into positive learning experiences for the students.

Excellent clinical teaching has been described as inspiring, supporting, and actively engaging with the students [1, 2]. There characteristics were described by students when referring to a small number of clinical teachers. They dedicated time for purposeful teaching and created a positive and supportive learning experience. Interestingly, they were also more likely to be seen as true ‘role models’ than those who predominantly aimed to teach by ‘role modeling’. In creating dedicated time for teaching and building rapport with students such teachers created positive experiences, both within and outside the purposeful teaching sessions. This correlates with what is known about excellence in role modeling in other settings [2, 3]. Having more assigned time for teaching and creating supportive learning experiences are some of the attributes of positive role modeling identified. Teachers who created positive experiences for students also demonstrated high standards of clinical competence, leadership, and humanistic personal qualities. They created positive gainful learning experiences and increased self-esteem and self-efficacy among learners. Self-efficacy, a belief about what one is capable of doing, is considered to be essential for adult learning [19]. Because in gauging self-efficacy students are able to self-assess their capabilities and translate skills into actions, which is an ultimate positive outcome to be achieved in experiential learning.

A significant determinant of students’ reactions to the teaching-learning experience was based on the attributes of the clinical teacher and the type of learning experience created by them. As described in social cognitive theory [20], individuals, their behaviors, and situations create strong influences affecting the way in which we react to these experiences. Strong influences such as feelings of fear, apprehension, and anxiety, can negatively affect learning experiences and its outcomes. ‘Figurative’ role models created negative teaching-learning environments by taking an authoritarian and hierarchical approach, similar to what was described by Paskins during his elective placement in a teaching hospital in Sri Lanka [15]. Students responded by avoiding or rejecting the negative interactions.

Hierarchical systems, haphazard instruction, and teaching by humiliation are not unique to the medical schools included here. Even though similar systems have been reported in other parts of the world [21–23], there are unique ways in which historical and contemporary systems of power create hierarchical education systems. South Asian cultures, as is the case in Sri Lanka, support hierarchical social structures demanding respect for elders, men, and people in positions of power. Teaching-learning environments are not immune to these archaic and regressive traditions and students are unable to escape feeling oppressed in this context [24]. Their negative reciprocal interactions appear to be a reaction to this situation.

The results of this study highlight the importance of making a distinction between role modeling and purposeful teaching. When students expect their clinical training to be predominantly in the form of purposeful teaching, and teachers believe ‘role modeling’ is synonymous with teaching, there is discordance between expectations and outcomes. Only a few clinical teachers were able to effectively navigate this duality in roles by manifesting positive professional characteristics while carrying out professional duties and also dedicating time for purposeful teaching.

Raising awareness among clinical teachers about their dual roles will enable them to distinguish between their different positions, and to better conceptualize, define, and organize their teaching approaches.

Faced with ever-increasing student loads in medical schools, clinical teachers are unlikely to have adequate time for dedicated teaching, role modeling, and clinical work. To overcome these barriers, purposeful teaching responsibilities could be delegated among designated and trained clinicians to cover a set of pre-determined learning objectives during organized teaching sessions. Role modeling should be an integral, and explicit part of clinical training during the final year’s training; it provides valuable opportunities to observe high standards of clinical care, professionalism, leadership, and humanistic personal qualities.

There appears to be an exigent need to shift from traditional didactic methods towards more interactive teaching strategies [1], such as demonstration, gradual release, and thinking aloud across all 3 medical schools. The value of strategic feedback and a repertoire of teaching-learning methods should be recognized. Students are known to be most receptive to feedback when they perceive an ongoing trusting relationship with their teachers, because feedback shared in this context is received as valid information for learning [25]. However, to build a mutually respectful trustful student-teacher relationship, broader systemic changes are needed to change the prevailing culture of teaching by humiliation and subordination.

The results of this study should be viewed in light of its limitations. Interviews with teachers and direct observation of the student-teacher interaction would have added a useful perspective to this study, however, given the confidentiality considerations, and the exploratory nature of this study, this was beyond its scope. We did not observe any differences in the experiences and/or the perceptions among the study participants from the 3 different medical schools. The cohesiveness of the experiences documented is noteworthy and is reflective of the culture and work practices across 3 medical schools in Sri Lanka at. However, as there have not been significant major curriculum revisions in these schools since the time of data collection in 2013 (personal communications^1^), the information presented in this paper could inform ongoing and/or future curricular revisions and reforms in Sri Lanka.

## Conclusions

This is the first in-depth study of medical students’ experiences during their final year clinical training in 3 medical schools in Sri Lanka. It highlights the importance of making a distinction between role modeling and purposeful teaching, for both students and teachers. In addition to clarifying the teachers’ roles, broader systemic changes are needed to address the prevailing culture of teaching by humiliation and subordination. Moreover, this study highlights the need for locally-relevant empirical evidence that can fil the knowledge gaps, inform academic practices, and guide medical education reforms in this region.

## Footnotes

1 Names not included to protect personnel and institutional identities
